# Assessment Methods for Service-Learning Projects in Engineering in Higher Education: A Systematic Review

**DOI:** 10.3389/fpsyg.2021.629231

**Published:** 2021-07-23

**Authors:** Marián Queiruga-Dios, María Jesús Santos Sánchez, Miguel Ángel Queiruga-Dios, Pedro Mauricio Acosta Castellanos, Araceli Queiruga-Dios

**Affiliations:** ^1^Accompaniment Institute, Universidad Francisco de Vitoria, Madrid, Spain; ^2^Sciences Faculty, Universidad de Salamanca, Salamanca, Spain; ^3^Education Faculty, Universidad de Burgos, Burgos, Spain; ^4^Civil Engineering Faculty, Universidad Santo Tomás, Tunja, Colombia; ^5^Research Institute on Fundamental Physics and Mathematics, Universidad de Salamanca, Salamanca, Spain

**Keywords:** service-learning, assessment, assessment tools, data collection, engineering, higher education

## Abstract

Service-learning (SL) helps engineering students to be involved in community activities and to be motivated by their studies. Although several reviews and research studies have been published about SL, it is not widespread in sciences and engineering at the university level. The purpose of this research is to analyze the different community services or projects where SL is implemented by engineering students and faculty and to identify the procedures that were usually implemented to assess SL-based courses and activities. Assessment could be considered as the evaluation of a specific module and the engineering competencies, the evaluation of the effectiveness of the SL program, the assessment of the participation of the student in those programs, and the assessment of whether students have achieved certain outcomes or gained specific skills. We conducted a systematic review with a search in three scientific databases: Scopus, Science Direct, and ERIC educational database to analyze the assessment methods and what that assessment covers. From 14,107 publications related to SL, 120 documents were analyzed to inform the conclusions of this study. We found that SL is widely used in several universities as experiential education, and it is considered an academic activity. The most widely used assessment technique is a survey to evaluate the engagement and attitudes of students and, to a lesser extent, teamwork presentations.

## Introduction

Undergraduate students in engineering usually do not have the opportunity to develop their personal and social competencies and skills during their studies at the university level, except when they undertake practices in companies. Students will need a solid technical background as well as educational experiences that could help them to develop a sense of responsibility, self-efficacy, professional skills (e.g., leadership, communication, team-building, critical thinking skills, and sense of civic responsibility), and outside-of-the-classroom skills among fellow students and among the community (Oakes and Thompson, 2004; Dennis and Hall, 2007; McCormick et al., 2008; Finsterwalder et al., 2010). The limited use of different methodologies in university contexts is usually due to the lack of time of supervisors and sometimes the absence of knowledge of other pedagogies (Abes et al., 2002; Andrews et al., 2005; Banzaert et al., 2006; Borkoski and Prosser, 2020). In this sense, service-learning (SL) could complement the training of the engineering student in the aspects and skills mentioned above.

The origin of SL can be attributed to the implementation of community service programs developed with the desire to accelerate the process of social evolution through the education of all people (Kenny and Gallagher, 2002). These cooperative education projects integrated real-world experiences into Antioch College studies in 1920 through an innovative set of learning and community-building strategies (Henderson and Hall, 1946). The USA government, under the authority of the Corporation for National and Community Service, implemented a program called Learn and Serve America (LSA) for K-12 and higher education institutions. LSA provides grants to support SL activities. The origin of SL is associated with a variety of government initiatives (Toncar et al., 2006). At the beginning of the twenty-first century, there have been numerous calls to reform engineering education in the USA to increase student understanding and engagement in society (Tucker et al., 2013).

The first study published in an international journal about SL dated 1950 (found in the Scopus search) was Simpson (1950), where SL was defined as “*learnings related to evaluation, record-keeping, resource getting and selecting, democratic discussion processes, and reading*” (p. 1). The widespread use of SL increased in the 90s. This pedagogy was defined by Jacoby (1996) as: “*Service-learning is a form of experiential education in which students engage in activities that address human and community needs together with structured opportunities intentionally designed to promote student learning and development. Reflection and reciprocity are key concepts of service-learning*” (p. 5). As a result of the different definitions and interpretations that have emerged and the different contexts and objectives, the National Service Learning Clearing House (2005), seeking a core concept, indicated that “Service-learning combines service objectives with learning objectives with the intent that the activity change both the recipient and the provider of the service. This is accomplished by combining service tasks with structured opportunities that link the task to self-reflection, self-discovery, and the acquisition and comprehension of values, skills, and knowledge content” (National Service Learning Clearing House, 2005).

Service-Learning is considered a pedagogical methodology, as an experiential educational practice, as a community service, as a social justice orientation, and as a philosophical worldview that combines academic learning with community-based activities to improve the realities where the service is performed and which considers who receives the service as a central element (Tsang, 2000; Butin, 2006; Derreth and Wear, 2021). SL is included in learning through service, which encompasses SL and extra-curricular activities such as Engineers Without Borders, Engineers for a Sustainable World, and Engineering World Health (Cooper et al., 2011; Bielefeldt et al., 2013). The final beneficiary of SL is the wider community; it is not volunteerism or charity and it is different from other types of community service because it is a course-based learning experience and includes clear learning objectives (Bringle and Hatcher, 1996; Oakes et al., 2002; Karayan and Gathercoal, 2005; Butin, 2006; Brand et al., 2019; Furco and Norvell, 2019). It encourages students to “think outside the technical box” (Bielefeldt et al., 2009, p. 14.873.10) and use their creativity (Swan and Veit, 2003). Thus, SL could be understood as an educational application of engineering principles and concepts through real-life community and service-based projects (Christensen and Yurttas, 2009). SL projects cover different projects, from domestic projects dealing with issues in a local community to large-scale international projects in developing countries (Sevier et al., 2012).

The Engineering Projects in Community Service (EPICS) program began in 1995 at the Purdue University in the USA (Coyle et al., 2005; Cummings et al., 2013; Zoltowski et al., 2014). The SL special issue in the Journal of Business Ethics gave SL a great impulse in 1996 (Kenworthy-U'Ren, 2008). Since then, the concept of SL has evolved. The proceedings of several annual conferences of the American Society for Engineering Education (ASEE) published articles on SL in engineering, and the American Association for Higher Education published a monograph that includes some results from engineering projects (Tsang, 2000). SL is used by instructors at all educational levels and disciplines. It is considered an instructional method that motivates and creates opportunities for students to apply what they learn during courses to real-world issues and helps students to understand course content better than using traditional research projects or even better than project-based learning (Cooper et al., 2011; Cooper and Kotys-Schwartz, 2013; Brand et al., 2019).

To implement a SL program, teachers identify the topics or contents of the curriculum that will be addressed, and they may even establish a theoretical framework of the activities (Kenworthy-U'Ren and Peterson, 2005). For example, university students did different activities, such as a real-world client-sponsored marketing project and integrated out-of-class experiences (Finsterwalder et al., 2010); interviewed community business owners and prepared an article for publication in the Chamber of Commerce newsletter (Arney and Jones, 2006); shared EPICS (Oakes et al., 2002); worked with students from health professions (Seifer, 1998); and developed ICT-based resources for pre-school and primary school students (Estrella et al., 2017).

To our knowledge, no systematic review has been conducted for SL in engineering education related to assessment processes. This study provides a systematic analysis of publications about SL in engineering studies in higher education and, more specifically, of the assessment methods that have been deployed.

The goal of this review is to know if SL is used as a learning approach in engineering education and how it is being assessed by academic instructors. For doing this, we answered the following research questions:

(1) What is the general character of the corpus in terms of categories of published articles about SL in engineering education?
(2) According to the bibliography analyzed, what are the main courses where SL is implemented in engineering degrees? As SL is sometimes part of the curricula, this could shape how these SL courses are evaluated.
(3) Which assessment tools are used to assess SL activities and outcomes?

The population considered in this systematic review is engineering courses; the intervention is SL in engineering; comparisons are methods and tools for assessing SL in engineering; the outcome is SL assessment tools in engineering; and finally, the context is higher education.

This review is structured as follows: “Methods” section details the search method and the procedures that were performed to conduct the systematic review and to get the results; “Results” section summarizes the results of the review, including answers to the research questions. Finally, “Discussion and conclusion” section includes a discussion of this study.

## Methods

We searched three different databases: Scopus, Science Direct, and Education Resources Information Center (ERIC). The ERIC database is the only one (from the three chosen) devoted entirely to education. We decided to add Scopus and Science Direct databases because there is currently a great number of faculty and researchers working in educational research and publishing articles indexed in these databases. The results showed that this election was appropriate.

The search was conducted for published journal articles, book chapters, conference articles, or dissertations until 2019 in the context of higher education and specifically related to engineering students.

**Figure 1:**
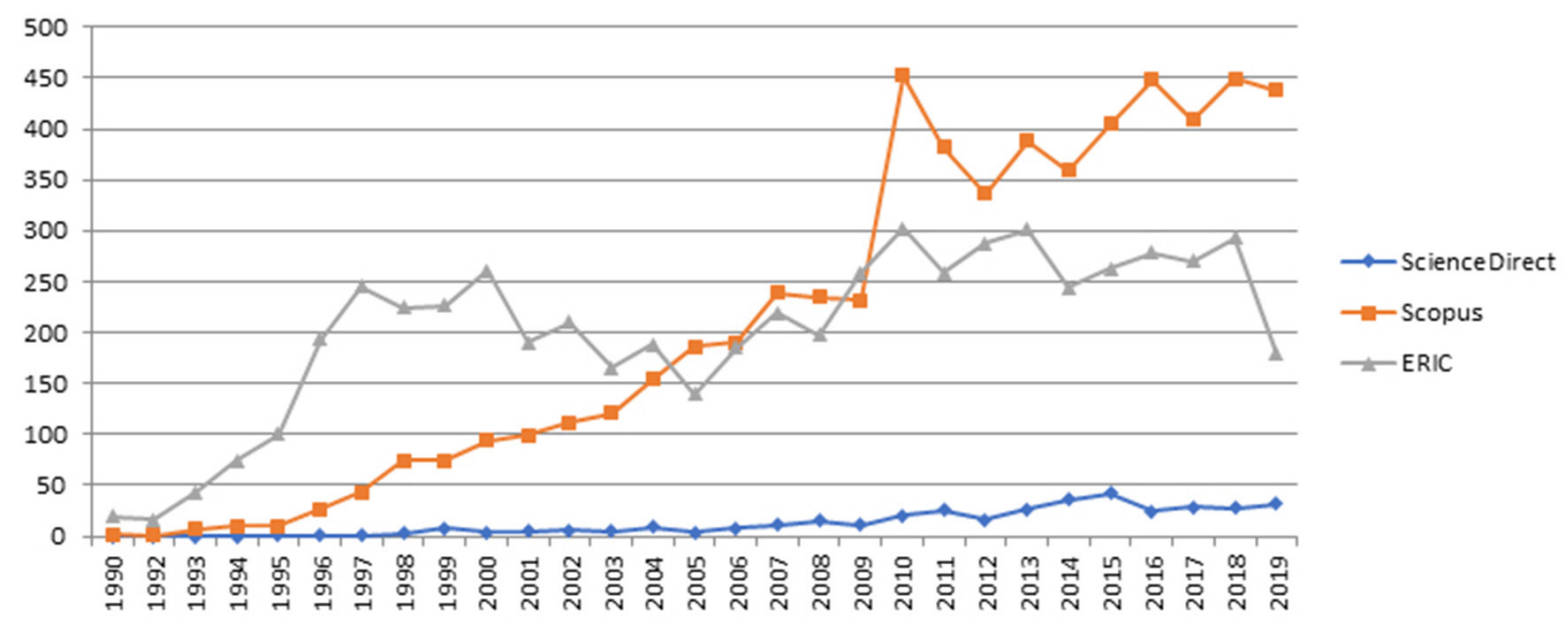
Graphical representation of the graph of the number of publications from 1990 to 2019, after searching for “service-learning” in Science Direct, Education Resources Information Center (ERIC), and Scopus databases.

Figure 1 shows the graph of the publications from 1990 to 2019, after searching only “service-learning” (in the title, keywords, and abstract) in the three databases. Results from Science Direct are not as relevant compared with the rest. We have not included other searching words such as community service and community engagement, because we wanted to center on the academic work where service is developed toward the community. The SL experience seeks to extend student learning beyond the training classroom, adapting the educational objectives to the needs of the communication partners. On the other hand, community service aims to improve reciprocal learning (Thomson et al., 2011).

With this initial search, we found that it is not common to develop SL projects in scientific, technological, and engineering areas; and it is even less common to use this pedagogy in engineering courses at universities. Thus, from 5,993 results related to SL in the Scopus database, most of the activities were done in social science areas (46.46%) and the remaining articles are distributed in the rest of the areas with a smaller percentage: engineering (9.25%), computer science (6.32), environmental science (1.15%), mathematics (0.85%), chemistry (0.59%), physics, and astronomy (0.17%).

**Figure 2:**
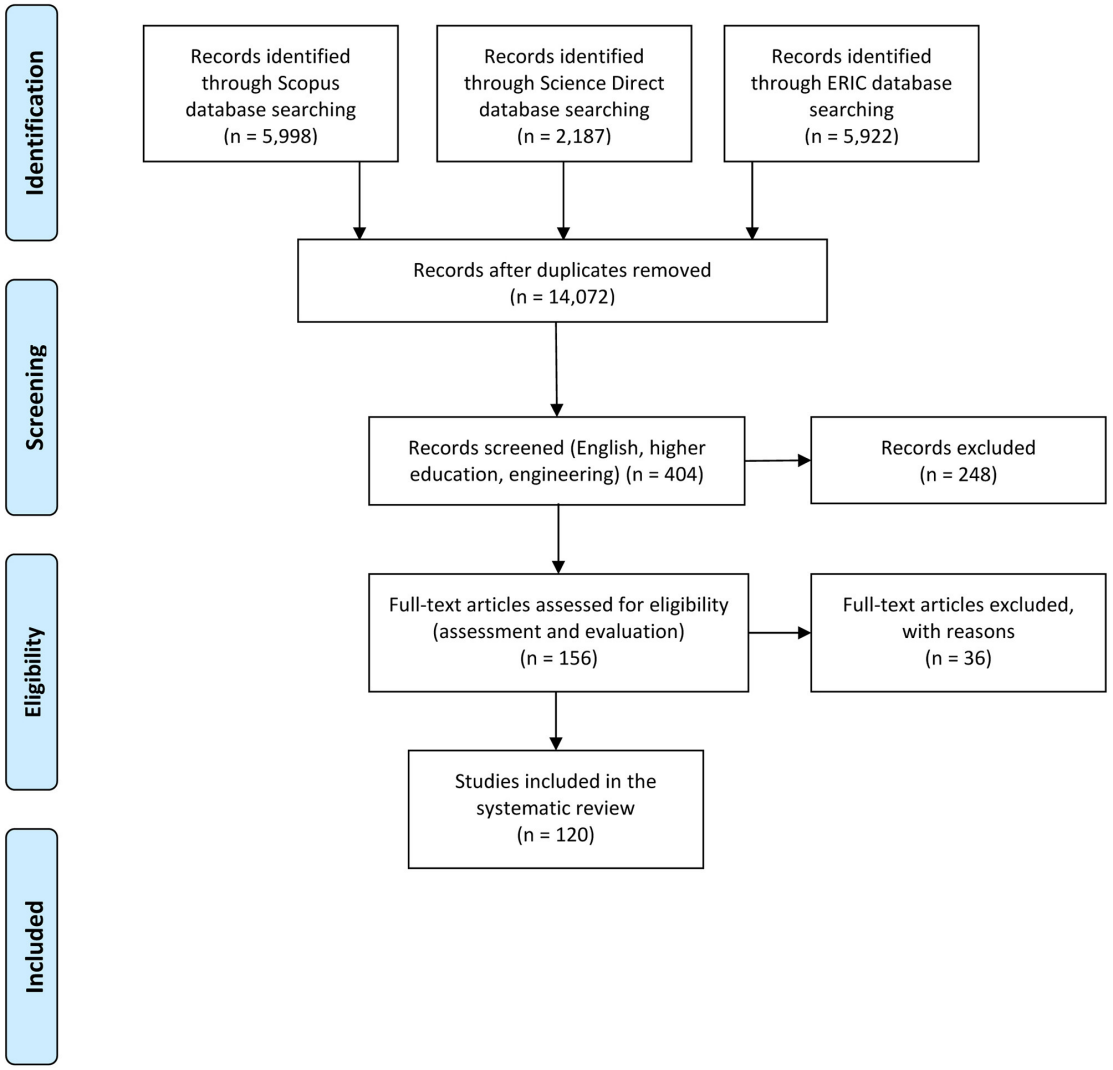
PRISMA flow diagram for the search developed during the review process (Moher et al., 2009).

The search flow conducted for this study is detailed in Figure 2. The inclusion criteria for this search were the following:

(1) The study considered SL articles in higher education and engineering studies.
(2) Non-English results were discarded because English is the most widespread in scientific publications.
(3) The study included results related to SL assessment.

After these inclusion criteria, the final number of articles is 156, where:

(1) English results about SL. Total number = 5,998 (Scopus) + 2,187 (Science direct) + 5,922 (ERIC) = 14,107.
(2) Results in higher education and engineering studies. Total number = 315 (Scopus) + 18 (Science direct) + 106 (ERIC) = 439. To develop the search, as each database has its own queries format, filters, and technical specifications, we restricted the subject areas to engineering, proceeding as follows:
  ⋆ In the case of Scopus, the search included the limitation to the exact keyword “Engineering Education” and subarea “Engineering.”
  ⋆ In the Science Direct database, the advance search included “engineering education” as title, abstract, or keywords.
  ⋆ For the ERIC database (through EBSCOhost), the search was done by adding “engineering education” as “DE Descriptors [exact].”
(3) We made an exhaustive search for assessment or evaluation in the title, keywords, and abstract. Finally, the total number of articles that were considered for the systematic analysis, without 35 duplicates, was Total number = 123 (Scopus) + 33 (ERIC) = 156.

From those 156 results, we conducted a new filtration considering the title and abstract contents. By doing this, 36 articles were discarded because they were based on one of the following:

- Conference proceedings book: Proceedings were not included because specific articles from them are already included in the results. The number of excluded results: 3.
- Disciplines that were not related to engineering: English pedagogy, second language learning, “Fill-in Worksheets” tool, microbiology, and future secondary school teachers. The number of excluded results: 5.
- Proposals were not directly linked to university students: SL activities targeted at K-12 educators and students, related to STEM disciplines. The number of excluded results: 7.
- Activities, courses, and studies that did not add value to the research questions, such as, internationalization activities at the university (not related to SL), faculty workshops on LTS, EWB (note related to engineering students or faculty), game-based learning, a reform of chemical engineering undergraduate curriculum, and general assessment of Accreditation Board of Engineering and Technology (ABET) professional skills (without the specific characteristics of SL). The number of excluded results: 18.
- Monographs and reviews general SL activities and experiential learning. These search results were related to general aspects such as SL pedagogy in engineering, SL reflection, and the institutionalization of SL, with no relation with assessment. The number of excluded results: 3.

After screening for inclusion, the final number of publications was 120. These publications are indicated with an asterisk in the References Section.

## Results

There is a great difference between the number of articles published about SL in different countries. Of 120 articles, 93% were published in the USA. This is the country where SL started and probably where this methodology is most prevalent. Moreover, 75.20% of the results were published in conference proceedings of the American Society of Engineering Education Annual (ASEE) Conference & Exposition. This conference is organized every year (since 1893) mainly in the USA, but sometimes in Canada.

We have also analyzed the authors of those articles, and we found that Bielefeldt participated in 14 publications, Swan in 13, Oakes in 10, Paterson in 9, Duffy in 7, Barrington and McCormick in 5 publications each, Karmer, Pierrakos, Thomson, and Zlotkowski in 4, Canney, Dewoolkar, Matson, and Tsang in 3, 35 authors participated in 2 publications and 233 authors only appear once.

The big difference in country allocation could lead to the conclusion that SL has been implemented and is being carried out as part of the university studies in some countries. It has become an institutional pedagogy. However, SL projects are developed in many other places. Thus, for example, the European Observatory of Service-Learning in Higher Education, in the 2019 Annual Report, analyzed active SL projects in different European countries. In this report, 11 SL projects developed from Erasmus+ or H2020 proposals were mentioned (Cayuela et al., 2020). On the other hand, in Latin America, it is common for projects to be developed outside the university environment, with most of the pioneering programs being developed in primary and secondary schools (Redondo-Corcobado and Fuentes, 2020).

The ABET criteria is another reason (maybe the most important) to include SL in engineering curricula in USA colleges, faculties, and universities. ABET EC 2000 set the following requirements for engineering universities (McCormick et al., 2008): (a) an ability to apply knowledge of mathematics, science, and engineering; (b) an ability to design and conduct experiments and analyze and interpret data; (c) an ability to design a system, component, or process to meet desired needs; (d) an ability to function on multi-disciplinary teams; (e) an ability to identify, formulate, and solve engineering problems; (f) an understanding of professional and ethical responsibility; (g) an ability to communicate effectively; (h) the broad education necessary to understand the impact of engineering solutions in a global and societal context; (i) a recognition of the need for, and an ability to engage in, life-long learning; (j) a knowledge of contemporary issues; and (k) an ability to use the techniques, skills, and modern engineering tools necessary for engineering practice. Taking into account that all accreditation systems [e.g., CDIO Proposal (Crawley, 2001; International Project Management Association, 2006), Tuning Project (González and Wagenaar, 2003), and Tuning Latin America, 2013] are in agreement (Queiruga-Dios et al., 2020), this interest in incorporating SL into the engineering curriculum could be extended to other nations.

From the 120 articles, 28 included ABET (129 times). Some articles that will be detailed later in this review defined different tools to measure if these (a)–(k) criteria are achieved by students during SL activities.

### General Character of the Corpus in Terms of Categories of Published Articles

In this systematic review, results were split into two different categories:

(1) General/SL results: General aspects of SL, including more theoretical aspects and reviews, deepening of SL pedagogy, and some results from analyzing experiences developed in different academic years (where the name of the project is not included in the article).
(2) Projects: This category included SL projects and the work that is proposed or developed for a specific project. This refers to literature that included specific studies about one or more projects. These articles included, in some cases, information about assessment methods.

Although most of the articles are related to specific SL projects, the main difference between both categories was that the first one does not include the name or title of the project.

General/SL results included, among others, the following topics: general views of Engineering for Developing Communities (EDC), which integrated social needs into the engineering courses and proposed new courses where SL was implemented (Bielefeldt et al., 2005; Ropers-Huilman et al., 2005; Duffy et al., 2007; Dukhan and Schumack, 2009; Green et al., 2009; Lucena et al., 2010; Hayden et al., 2011; Vernaza et al., 2012; Whitman and Mason, 2013; Balascio, 2014; Hayford et al., 2015; McLean et al., 2018); the technology integration framework for SL (Salam et al., 2019); studies to analyze the impact of the experience on students, faculty, and/or the affected communities (Mehta and Enger, 2004; Bauer et al., 2005; Zoghi and Pinnell, 2005; Banzaert et al., 2006; Schaffer et al., 2007; McCormick et al., 2008, 2010; Duffy et al., 2009; Huyck et al., 2009; Swan and McCormick, 2009; Paterson, 2010; Wiggins et al., 2011; Reynaud et al., 2013; Love et al., 2014; Armstrong et al., 2019); attitude toward SL between male and female students (Tsang, 2001; Thompson et al., 2005; Tucker et al., 2013; Lens and Dewoolkar, 2015); analysis of ethics, civic, and social responsibility (SR) attitudes through SL (Williams, 2002; Zoltowski et al., 2013; Bielefeldt and Canney, 2014) and classroom discussions and critical reflection articles integrated into the ABET assessment plan (Newbolds et al., 2017); specific programs, such as EPICS program, designed for the integration of undergraduate engineering students from different engineering disciplines and from different educational levels, and SL incorporation throughout a College of Engineering (Service-Learning in Civic Education, SLICE) from University of Massachusetts Lowell, which is integrated into the mandatory first-year curriculum and has as its goal to serve as an introduction to engineering design for freshmen with limited technical backgrounds in engineering (Oakes et al., 2001; Immekus et al., 2005; Dutta and Haubold, 2007; Burack et al., 2008; Foster and Spivey, 2012; Bielefeldt et al., 2013; Cummings et al., 2013; Underwood, 2013); and others.

The remaining 52 results were part of the Project category. They included the implementation, development, and experiences in specific projects such as projects for school students (Wang et al., 2012; Ansari et al., 2013) or in local historical society (Douglas, 2017); engineering projects (Duffy et al., 2008; Birdsong, 2012; Najmr et al., 2018); and outreach projects (Ocif and Marshall-Goodell, 1996). Several projects were developed related to sustainability or environmental protection, for the community, or to improve the quality of life in several different contexts and countries (Christensen and Yurttas, 2009; Hayden et al., 2010).

We found a few projects directly related to engineering curriculum: more shop floor operations and lean manufacturing that are difficult to teach in a classroom setting (Miles et al., 2005); projects in three different technology companies of varied size and within diverse product sectors (Stockman et al., 2017); and a local infrastructure report card to increase awareness of the infrastructure (Roberts et al., 2007).

This list of projects is quite long as suggested by Dennis and Hall (2007), who claimed that “*One of the most critical tasks associated with service learning is selecting an appropriate project with the correct magnitude and technical complexity that will insure success in the achievement of the outcomes of the program*” (p. 5). Any of these published projects could give an idea to a reader about a new proposal for addressing SL activities.

A table with detailed information about all the search articles is included in Supplementary Material.

### Main Courses Where SL Is Implemented in Engineering Degrees

The most common curricular model for SL in the reviewed literature was implemented in first-year courses as part of engineering introductory courses. However, SL is also commonly used in senior courses and, to a lesser extent, in the second and junior courses (nine results showed the use of SL in courses of graduate students). From the search result, 114 articles contained information about the level of students and 21.93% were related to all undergraduate years, 28.5% to first-year students, 10.53% to sophomore, and the same percentage to junior students, 17.54% corresponded to senior students, and the remaining 10.53% are projects developed by graduate students.

The way in which SL is implemented is through a curricular approach, cocurricular activities, SL-based courses, or also through extracurricular activities or SL programs, such as SLICE, or Engineers for a Sustainable World (ESW) project. When SL is integrated into existing courses or new courses are defined to implement this pedagogy, we consider a curricular approach. Cocurricular activities may be outside of class time, including the development of a capstone project or a research proposed by a teacher. Finally, extracurricular projects mean activities that may or may not be linked to the studies. Although SL is an activity integrated into credit-bearing courses, in many cases, projects can pass between curricular and extracurricular activities (Oakes, 2004; Bielefeldt et al., 2009).

Several introductory courses implemented SL as innovative pedagogy. These included Introduction to Engineering (Meadows and Jarema, 2006; Kazmer et al., 2007; Dimitriu and O'Connor, 2008; El-Gabry, 2018), Introduction to Engineering Design (Zoghi and Pinnell, 2005; Dutta and Haubold, 2007; Bernardoni et al., 2009), Introduction to Mechanical Engineering (Tsang et al., 1996), Introduction to Materials Engineering Design I, II, and III (Harding et al., 2010), Introduction to Civil Engineering and First-Year Engineering Projects (Bielefeldt, 2006a), Introduction to Engineering I, and Community-based Engineering Design Project I and II (Reynaud et al., 2013). Apart from these, there were other engineering courses where SL was used and is being used. Some of them are Civil Engineering, Materials Science and Industrial Engineering (Ansari et al., 2013), Environmental (Bielefeldt, 2006a), Chemistry (Najmr et al., 2018), Renewable Energy Engineering (Gleixner et al., 2011), or Studio and Laboratory courses (Cowan et al., 2013). Capstone senior design courses (Bielefeldt et al., 2007; Dennis and Hall, 2007; Lens and Dewoolkar, 2015) were also part of these sets of disciplines where SL is implemented.

After this detailed information about the courses in which SL is implemented in engineering, there was no course where SL could be considered the most frequently used pedagogy. It was usually implemented in different courses, but it was least common in core engineering science courses or specialized courses in upper levels. SL was considered most appropriate for design courses (Bielefeldt et al., 2012). Nevertheless, in some SLICE programs, SL was integrated into core courses, and it involved engineering theory, methods, and skills, such as statistics, thermodynamics, heat transfer, fluids, circuits, or dynamics (Duffy et al., 2009). From the search results, 61.90% corresponded to existing courses, 19.05% to SL programs such as EPICS or SLICE, 9.52% to cocurricular components, such as capstone projects or other research, and the remaining 9.52% were related to new SL courses.

### Assessment Tools to Assess SL Activities

Like all educational contexts, SL assessment is a vital activity to evaluate the quality of student learning and to determine the learning outcomes acquired by students, including their engagement and improvement. Moreover, in the case of SL, the ways of learning do not allow traditional modes of assessment, such as tests or quizzes (Cummings et al., 2013). Within engineering education, SL is generally conducted *via* PBL; thus, this approach is often referred to as project-based service-learning (PBSL) by its practitioners (Bielefeldt et al., 2009). Written reports, interviews with students, surveys, a portfolio, or a multimedia presentation are some of the assessment tools usually used for PBL (Frank and Barzilai, 2004). Assessment activities have been used to measure the impact of SL on students, faculty, and the community (less) and also to measure the impact of specific projects and their contributions to the community (Bielefeldt, 2006b). Assessing the SL activity from the point of view of the student, as SL is a pedagogical tool (Toncar et al., 2006), is most important. Assessment methods have been more commonly conducted in course-based SL than cocurricular/extracurricular SL activities. When analyzing assessment methods and assessment results, the nature of the course must be considered. They are different in compulsory or not compulsory or curricular or cocurricular courses. Assessment of student learning in extracurricular projects may lead to greater integration of these activities into credit-bearing courses (Bielefeldt et al., 2009).

One of the techniques most commonly used to assess SL activities was written surveys to get the opinions of students, to get the interests of faculty, and to improve SL programs. These surveys provided a different type of information that varied greatly in length, complexity, time to be conducted, previous use in engineering education, or other contexts (Banzaert et al., 2006; Bielefeldt, 2006a; Bielefeldt et al., 2009). Some of the surveys that were used in SL in engineering studies were:

- Community Service Attitudes Scale (CSAS) (Bauer et al., 2005).
- Survey-based on ABET/National Academy of Engineering (NAE) criteria with additional open-ended questions (Ansari et al., 2013).
- Engineering Professional Responsibility Assessment (EPRA) (Bielefeldt and Canney, 2014).
- The STAR (situation, task, action, and result) method of behavioral interviewing (Balascio, 2014).
- Service-learning Benefit (SELEB) scale (Toncar et al., 2006).
- The academic profile of the Educational Testing Service (Mehta and Enger, 2004).
- Cooperative Institutional Research Program's (CIRP) freshman survey, and the “Your First College Year” (YFCY) survey from the Higher Education Research Institute (HERI), and the BarOn Emotional Quotient inventory, which are implemented to assess leadership (Mehta and Enger, 2004).
- The Draw an Engineer Test (DAET) (Portsmore and Swenson, 2012), which focuses on the responses of students who answer the question “What does an engineer do?” (Knight and Cunningham, 2004).
- Cross-Disciplinary Functioning survey (CDFS) (Schaffer et al., 2010).
- Other surveys were conducted before (or in the middle) and at the end of the SL project (Birdsong, 2012). Some of these surveys include identical pre- and post-test questions (Brand et al., 2019).
- Classroom Climate inventory (Tsang et al., 1996).
- The Michigan Organizational Assessment questionnaire (Tsang et al., 1996).
- Job Satisfaction questionnaire (Tsang et al., 1996).
- The Engineering Ethical Reasoning Instrument (EERI) (Zoltowski et al., 2013).

To summarize, 63 articles out of 120 have used surveys (questions or questionnaires) to assess SL activities. So, this is the most widely used method for this purpose.

Some other methods that have been used to assess SL were:

- To write a short or a long final project report made by students summarizing their experience. This was used to improve and reinforce the written communication skills in engineering education (Bielefeldt et al., 2009; Birdsong, 2012; Balascio, 2014).
- Ten minutes short interview (Bielefeldt et al., 2012).
- Presentation of a poster where students compared their results with all of the other teams and were asked to explain technical aspects of the project (Birdsong, 2012).
- Preparation and presentation of a report and oral presentation to the class and community partner. Additionally, the students provided the community partner with materials, data, and final reports (if applicable) (Brand et al., 2019).

The assessment tools were analyzed related to the outcomes of students (as shown in Table 1). The assessed features were recruiting/retention/diversity (R), post-educational professional performance (P), technical (T) and non-technical skills (S), student knowledge (K), and attitudes (A). We found that 86 articles out of 120 included the learning outcomes that were measured by the assessment tools in students. The most measured outcomes were, in the order, A and S with 27.04 and 26%, respectively, and then T and K with 15.45 and 12.88%, respectively, and finally R with 9.44 and P with 8.58%.

**Table 1:** Search results related to surveys (SV), reports (RP), and Presentations (PT) related to the learning outcomes that are measured (R, recruiting/retention/diversity; P, post-educational professional performance; T, technical skills; S, non-technical skills; K, knowledge; or A, attitudes).

	**R**	**P**	**T**	**S**	**K**	**A**
SV	16	14	22	43	19	50
RP	3	4	6	11	6	8
PT	3	3	4	7	2	5

Assessment processes usually included student self-assessments of achievement of learning objectives, summative assessments where students indicated some of the most valuable outcomes they learned, and the level of satisfaction of project partners (Bielefeldt et al., 2009).

Several studies did not include the assessment technique that was used to get results. But these results and sometimes the consequences were included in those articles (Bielefeldt et al., 2007).

On the other hand, students participating in an SL program (curricular or non-curricular, for freshmen, sophomore students, or as part of a capstone design course), no matter the type of activity, were evaluated via standard grading (written exams, reports, presentations, or any other technique) (Bielefeldt et al., 2009). In almost all cases, the assessment methods that were used with students participating in SL activities included the assessment of the knowledge, non-technical skills, technical skills, attitudes, recruiting/retention/diversity, or post-educational professional performance (Bielefeldt et al., 2009), but all of them from the point of view of the student, i.e., without theoretical questions about course curricula. We could conclude, as was established by Toncar et al. (2006), that “*no effective instruments presently exist to measure students' perceptions of the benefits of service-learning*” (p. 226).

Rubrics were used in several SL projects to ensure that the students understand and meet the requirements of each module. Students presented midcourse and end-of-course reports and participated in questionnaires with these, and they received feedback from the instructor and community partners before the conclusion of the project (Carducci, 2014; Brand et al., 2019).

Structural equation modeling was used by Levesque-Bristol et al. (2011) to examine the effectiveness of SL. They used surveys to analyze the learning climate and positive forms of motivation, civic skills, problem-solving, and appreciation of diversity across more than 30 academic courses involving more than 600 students.

## Discussion and Conclusion

This study has presented a systematic review about SL in engineering education, and specifically the way to assess SL activities. As far as we know, this review has not been done before.

During SL activities, it is important to integrate the course objectives with the service objectives and goals. As was mentioned in some published articles, SL has demonstrated to be a feasible way for improving professional skills and integrating non-technical and non-academic areas into undergraduate engineering courses (Oakes et al., 2002).

According to the categorization carried out on the corpus of the article, although there was a large number of articles included in the General SL category (which included articles on theoretical aspects, reviews, and ideas about pedagogy), a significant number of articles (around 44%) were related to the planning, design, and development of SL specific projects in different countries and communities. The use of SL in higher education and in engineering courses is increasing. There is a great difference between the number of articles published about SL in different countries. The USA is, so far, the country where SL is most widespread. This is related, presumably, to the interest in the publication of the research carried out regarding the SL projects. Some universities have established SL as a curricular methodology in several courses with specific programs, such as the EPICS program (Coyle et al., 2005; Cummings et al., 2013; Zoltowski et al., 2014) and SLICE (Duffy et al., 2009). However, as indicated, important SL projects have also been developed in other European (Cayuela et al., 2020) or Latin American (Redondo-Corcobado and Fuentes, 2020) countries, for example. The increasing use of SL pedagogy in engineering is due to the importance of acquiring social, professional, civic, and human competencies. This need for service to society was also included in the approval of the 2030 Agenda for Sustainable Development and the promotion of the improvement of the living conditions of humanity. New social and economic theories seek more moderate capitalism based on the common good, and for that to happen, it is necessary for the new generations to be socially responsible in the search for well-being and the sustainability of the planet through their professional development. Moreover, the value of the community is increasing with theories such as communitarianism, where members are responsible for the well-being of the rest of the members (Etzioni, 1996).

Detailed information about all the search results is included in Supplementary Material.

Regarding the development of SL projects, it was found that many of them have been implemented in engineering courses, from freshmen to graduate students. The level where SL was most extensive was in first-year courses, but it is also used in all undergraduate courses and capstone courses, where students develop research about their SL projects, and in graduate courses. SL serves as an introduction to engineering for first-year students with limited technical backgrounds. Furthermore, these activities favor the engagement of students and reduce dropout rates of engineering studies. SL in the first year can provide a basis for an engineering program building upon those early experiences in later courses.

On the other hand, from the 120 articles analyzed during this systematic review, 83 included the type of SL that was carried out regarding the course or curricular characteristics.

Finally, concerning the assessment activities, most used surveys (68.13% of 91 articles that include information about the assessment tools). Surveys allowed assessing students (75% of 98 articles), academics (8% of 98), and community partners (15% of 98) outcomes, although these last two actors were rarely taken into consideration in assessment processes. In the case of students, the assessment impact analyzed student knowledge, technical and non-technical skills, attitudes, recruiting/retention/diversity, and post-educational professional performance.

From these results, we can conclude that most of the assessment systems did not include technical (engineering) aspects of the courses. Only 29.9% of the assessment tools reported were not surveyed. Although SL is considered a curricular activity, technical aspects of the SL projects were not reported because technical aspects are assessed out of SL activities. No article in the literature reported using an exam to assess knowledge and skills acquired in the SL program, because these search results showed the outcomes of SL and not their relationship with the rest of engineering topics and courses.

As future research, we plan to study university websites and analyze whether they incorporate information about SL and what type of information they include. It is common to use websites and social networks while implementing SL activities and before publishing results. Some higher education institutions participate every year in local, national, or international projects where SL projects are being developed. This is promoting the use of SL.

Moreover, several countries have different associations for SL, such as the “European Observatory of Service-Learning in Higher Education” in Ireland^1^, “*Red Española de Aprendizaje-Servicio*” in Spain^2^, and “CLAYSS, *Centro Latinoamericano de Aprendizaje y Servicio Solidario*”^3^ in Latin America. These associations include students and faculty from different disciplines. This will improve the quality of education through interdisciplinary and multidisciplinary works.

Finally, the authors want to express that educating engineering students with the SL pedagogy is investing in a better future for society and humanity. This was the idea of the first community service programs developed by the universities.

To this date, there are no studies like the one presented in this study, which is a relevant topic for engineering education, because project development is a key part of the engineering curriculum. A deepening in the experiences of already completed and ongoing SL projects and the assessment mechanisms of students will allow the implementation of this pedagogy in more universities. The limited use of active methodologies in university contexts is not very widespread. This is usually due to the lack of time of the faculty, the absence of knowledge of other pedagogies, and their benefits for the integral training of the student. In this sense, the SL approach could complement the training of the engineering student in the aspects and skills mentioned above.

## Data Availability Statement

The original contributions presented in the study are included in the article/Supplementary Material, further inquiries can be directed to the corresponding author/s.

## Author Contributions

MQ-D, PA, and AQ-D have contributed to the initial part of the documents search, the analysis of data, and results. MS and MÁQ-D contributed to the methodology and discussion. All the authors participated in the document elaboration and revision of the article.

## Conflict of Interest

The authors declare that the research was conducted in the absence of any commercial or financial relationships that could be construed as a potential conflict of interest.

## Publisher's Note

All claims expressed in this article are solely those of the authors and do not necessarily represent those of their affiliated organizations, or those of the publisher, the editors and the reviewers. Any product that may be evaluated in this article, or claim that may be made by its manufacturer, is not guaranteed or endorsed by the publisher.

## Footnotes

^1^Available online at: https://www.eoslhe.eu/service-learning-in-ireland/ (accessed November 13, 2020).

^2^Available online at: https://www.aprendizajeservicio.net/que-es-el-aps/ (accessed November 13, 2020).

^3^Available online at: http://www.clayss.org.ar/ (accessed November 13, 2020).
