# Supplementary material for: Assessment Methods for Service-Learning Projects in Engineering in Higher Education: A Systematic Review
Source: Front Psychol. 2021 Jul 23;12:629231. doi: 10.3389/fpsyg.2021.629231 (PMC8343223; doi:10.3389/fpsyg.2021.629231)
Supplement: Supplementary file 1 [file Data_Sheet_1.docx]

Supplementary Material

# Detailed description of search results

This Supplementary material includes a table with detailed information of all the 120 papers. This summary contains specific information about the following topics:

1. Type of service learning that was carried out regarding the course or curricular characteristics (Duffy et al., 2000) were separated into: existing courses (T1), co-curricular components (T2) such as capstone projects or other research, new service-learning courses (T3), and service-learning programs (T4) such as EPICS or SLICE.
2. Level of students: First year (L1), Sophomore (L2), Junior (L3), Senior (L4), Graduate (L5), or All undergraduate years (L6).
3. Discipline or course into which the SL is developed.
4. Program: Most of the times, to be part of a program excludes to be part of a course, and vice versa.
5. Assessment method that is mentioned in the paper: usually survey, presentation or written report.
6. Impact of the assessment related to what are the learning outcomes that are measured by the assessment tools (Bielefeldt et al., 2009; Jeffers et al., 2014): knowledge (K), non-technical skills (S), technical skills (T), attitudes (A), recruiting/retention/diversity (R), or post-educational professional performance (P).
7. Assessed group: students, faculty, community, or others.
8. Service-learning project.

A white (empty) cell indicates that information is not specified.

Some papers that are written in too general terms or included in the category General / SL in Results Section, were kept in the table with only the corresponding explanation. In some cases, there is not too much information about levels, courses, programs or projects, but they include additional information about the assessment method. In this case, as all papers, we included the information that is available in the paper.

|  | Reference | Type of SL | Level of students | | Discipline or course | Program | | | | Assessment method | | Impact | Assessed group | | | | SL project |
| --- | --- | --- | --- | --- | --- | --- | --- | --- | --- | --- | --- | --- | --- | --- | --- | --- | --- |
| 1 | Ansari, 2013 | T1 | L1 | | civil engineering, materials science and industrial engineering |  | | | | survey | | A | Students | | | | K-12 service learning project at a science museum / outreach teaching |
| 2 | Armstrong et al., 2019 | This study is centred on measuring the impact of SL programs on the affected communities. | | | | | | | | | | | | | | | |
| 3 | Balascio, 2014 | T2 | | L4 |  |  | | | | Reflective Journal  Final report  Survey: STAR method | | K, S, A | Students,  Supervisors (survey) | | | | Not specified |
| 4 | Banzaert et al., 2006 | several | | L6 | General / SL results |  | | | | surveys | | S, A | Students,  Faculty | | | | Several |
| 5 | Bauer et al., 2005 | T4 | | L2 | Before using SL methodology | Humanitarian Engineering | | | | survey^^[[1]](#footnote-1)^^ | | A | Students,  Faculty | | | | Not specified |
| 6 | Bernardoni et al., 2009 | T1 | | L5, L6 | Introduction to Engineering  Design course (between others) | OLPC | | | |  | |  |  | | | | One Laptop Per Child Project |
| 7 | Bielefeldt, 2006a | T1 | | L4, L1 | L4: Environmental  L1: Introduction to Civil Engineering & First Year Engineering Projects | Engineering for Developing  Communities (EDC) | | | | Survey | | S, A | Students | | | | Filtrón drinking water filter and other EDC projects |
| 8 | Bielefeldt, 2006b | T2, T4 | | L5, L6 |  | Several^^[[2]](#footnote-2)^^ | | | | Survey^^[[3]](#footnote-3)^^ | | S, A | Students | | | | Filtrón and others |
| 9 | Bielefeldt and Canney, 2014 |  | | L1 |  |  | | | | Pre and post-Survey^^[[4]](#footnote-4)^^ | | S, A, P |  | | | |  |
| 10 | Bielefeldt et al., 2005 | Proposal of a EDC program and results from a workshop on “Integrating Appropriate-  Sustainable Technology and Service-Learning in Engineering Education” | | | | | | | | | | | | | | | |
| 11 | Bielefeldt et al., 2007 | T3 | | L4 | A capstone design course: Engineering for the Developing World (EDW) |  | | | | Different assessment methods^^[[5]](#footnote-5)^^ | | K, S, T | Students | | | | International engineering projects in Rwanda and Mexico. |
| 12 | Bielefeldt et al., 2009 | T1, T2, T4 | | L6 | Several courses | EPICS, SLICE, | | | | Report (assessed with a rubric) | | ALL | Students | | | |  |
| 13 | Bielefeldt et al., 2012 | Analysis of faculty engagement through a survey during a summit | | | | | | | | | | | | | | | |
| 14 | Bielefeldt et al., 2013 | T2 | | EPICS , Review of LTS | | | | | | | | | | | | | |
| 15 | Birdsong, 2012 | T1 | | L1, L2 | Engineering Dynamics course |  | | | | Survey (mid and end of the course) | | K, S, A | Students | | | | Automotive safety for high-school students |
| 16 | Brand et al., 2019 | T3 | | L6 | Hazards, vulnerability, and risk |  | | | | Report, oral presentation, survey | | S, A, R, T | Students | | | | Hazards, vulnerability, and risk projects |
| 17 | Burack et al., 2008 | T1 | | L6 | Several courses | SLICE | | | | Interviews, Surveys | | K, A, P | Faculty | | | | projects to benefit some entity associated with the city |
| 18 | Canney and Bielefeldt, 2012 | T1 | | L6 | Introductory classes, environmental engineering fundamentals class, civil engineering senior capstone design and structures and community development classes. |  | | | | Survey^^[[6]](#footnote-6)^^ EPRA | | S, A, R, P | Students | | | |  |
| 19 | Chan, 2012 | Literature review on the diverse forms of assessment currently used for community service types of experiential learning in higher education | | | | | | | | | | | | | | | |
| 20 | Chrispeels et al., 2019 | T1^^[[7]](#footnote-7)^^ | | L1 | Biology and the Human Condition |  | | | Pre- post-surveys | | | K, S | Students | | | Genetically modified crops and their use in agriculture for high school students (peer teaching) | |
| 21 | Christensen and Yurttas, 2009 | T1 | | L2 | Introductory material and energy balances course |  | | | Surveys, oral and written report | | | S, T, A, R, P | Students | | | Habitat for Humanity: to design an affordable green home, and local recycling methods | |
| 22 | Cooper et al., 2011 | T1 | | L3 | Component Design |  | | | Pre-course and post-course surveys | | | K, S, T | Students | | | Bicycle trailer and hitch system for homeless | |
| 23 | Cooper and Kotys-Schwartz, 2013 | T1 | | L3 | Component Design |  | | | Pre-course and post-course surveys | | | K, S, T | Students | | | Adapted Tricycle Project for children and young adults in the community who have physical disabilities | |
| 24 | Cowan, 2008 | T1 | | L6 | Architectural Technology Program courses |  | | | Survey^^[[8]](#footnote-8)^^ | | | A | Community partners | | |  | |
| 25 | Cowan et al., 2013 | T2 | | L4, L5 | Studio and laboratory courses |  | | | Survey^^[[9]](#footnote-9)^^  Final report | | | S, A | Students and Community partners | | | The reuse and design of aging trains to develop housing and work environments in a specific community | |
| 26 | Cummings et al., 2013 | T4 | | L6 |  | EPICS | | | Survey  With Rubric for students | | | K, S, T, A, P | Students and Community partners | | | Several projects | |
| 27 | Dennis and Hall, 2007 | T1 | | L4 | Senior Design course |  | | | survey | | | S, A, P | Students | | | A timber bridge for a non-profit educational organization located about 45 miles from campus | |
| 28 | Dimitriu and O'Connor, 2008 | T1 | | L1 | Introduction to Engineering course |  | | | A report and a presentation^^[[10]](#footnote-10)^^,  a journal with daily activities, written reports | | | S, P | Students | | | Work on foundations,  another team on plumbing, another team on electrical wiring, and so on | |
| 29 | Ding, 2008 | T1 | | L2 | Interior design course |  | | | A reflective journal, a written exam.  Rubrics | | | K, S, T, |  | | | To design a single  family house for Habitat for Humanity with an ADA accessible bath and kitchen^^[[11]](#footnote-11)^^ | |
| 30 | Dixon, 2010 | This paper is related to promotion and tenure policies in Engineering and  Technology Programs within the USA. | | | | | | | | | | | | | | | |
| 31 | Douglas, 2017 | T1 | | Introductory engineering classes’ paper that compares a traditional class at a brick-and-mortar campus and the same class offered via the Online Campus. | | | | | | | | | | | | To work with a local historical society to create interactive museum exhibits and other projects | |
| 32 | Duffy et al., 2007 | T1 | | L6 | Introduction to engineering  design lab, dynamics, convective process, statics, machine elements, laboratory course, and several capstone course projects | SLICE | | | Surveys and interviews of faculty,  students, and community partners and student reports and presentations | | | S, A, R, P | Students, faculty and Community Partners | | | Designed and built educational displays for the local national historic park  device development for student relatives or friends with disabilities, and several other projects | |
| 33 | Duffy et al., 2008 | T1 | | L6 | 35 different undergraduate required courses |  | | |  | | |  |  | | | Several projects | |
| 34 | Duffy et al., 2009 | T1 | | L6 | introduction to engineering,  kinematics, heat transfer, fluids, circuits, microprocessor, etc. courses | SLICE | | | Surveys | | | S, R, P | Students and faculty | | | Build various parts of an automated canal lock opener for a local national park, and several other projects | |
| 35 | Dukhan and Schumack, 2009 | Service learning as a new pedagogy | | | | | | | | | | | | | | | |
| 36 | Dutta and Haubold, 2007 | T1 | | L1 | Introduction of engineering design | SLICE | | |  | | |  |  | | | Projects of five genres: (1) assistive devices, (2) civil/architectural designs, (3) educational tools, (4) information technology, and (5) urban development | |
| 37 | El-Gabry, 2018 | T1 | | L1 | Anatomy & Physiology I | Project Prize: A New York State Liberty Partnerships Program | | | Surveys | | | S, A, R | Students and community partners (and K–12 students) | | | ASL (Academic Service-  Learning) K–12 outreach program about homelessness | |
| 38 | Fang and Pines, 2007 | T1 | | L4, L5 | Transportation-related courses, such as Transportation Engineering I and Transportation Engineering II |  | | | Surveys and  public presentation | | | K, S, T | Students | | | To evaluate traffic operations along an important urban corridor and propose improvement alternatives | |
| 39 | Foster and Spivey, 2012 | T1 | | L2 to L4 | Servant Engineering | EPICS-based | | | Surveys^^[[12]](#footnote-12)^^ and written reflections | | | K, S, T, A, P | Students | | | Projects fits into the following tracks: education outreach, community service, appropriate technology for overseas, and assistive technologies | |
| 40 | Gilbert, 2014 | This paper is related to collaboration between a school of social work and a school of engineering and analyzes the collaboration using a model of interdisciplinary social work. | | | | | | | | | | | | | | | |
| 41 | Gleixner et al., 2011 | T1 | | L3, L4, L5 | Renewable Energy Engineering |  | | | survey | | | S, A | Students | | | Bring renewable energy technology to an afterschool program for 4th- 7th graders at a nearby community centre. | |
| 42 | Gleixner et al., 2013 | T4 | | L1 | A ten day residential summer transition program | EXCEED^^[[13]](#footnote-13)^^ | | | survey | | | S, A, R | Students | | | To build something for a local non-profit agency. | |
| 43 | Goggins, 2012 | Assessment method and results are not specified | | | | | | | | | | | | | | | |
| 44 | Green et al., 2009 | T1 | | L4 | Design and Prototyping class |  | | | survey | | | K, S, T, A | Students | | | Fully functional prototypes to local “customers” with physical disabilities. | |
| 45 | Harding et al., 2010 | T1 | | L1 | Introduction to Materials Engineering Design I, II, and III |  | | | survey | | | S, A, R | Students | | |  | |
| 46 | Hayden et al., 2010 | This paper presents a brief overview of the changes made during our department level reform process | | | | | | | | | | | | | | | |
| 47 | Hayden et al., 2011 |  | |  | This paper presents a brief overview of the changes made during a department level reform process | | | | | | | | | | | | |
| 48 | Hayford et al., 2015 | T1 | | L6 | Speech |  | | | STEMSL tool (Tool for Evaluating STEM Literacy) | | | K, S, T, A | Students | | Creation of digital audio files providing content on an assigned specimen displayed in a natural history museum for regional K-12 students and casual visitors to the museum. | | |
| 49 | Huyck et al., 2009 | This paper is about reflective thinking levels produced by involvement in service-learning projects | | | | | | | | | | | | | | | |
| 50 | Immekus et al., 2005 | T4 | | L6 |  | EPICS | | | Survey/self-report measures^^[[14]](#footnote-14)^^ | | | S, A |  | |  | | |
| 51 | Jansson et al., 2005 |  | | L3, L4 | Engineering Clinics |  | | |  | | |  |  | | To site, design, permit, and install photovoltaic (PV) systems around campus | | |
| 52 | Jeffers et al., 2014 | T4 | | L3, L4, L5 | Not specified |  | | | Reflective journals | | | S, T, A | Students | | The design and construction of a pedestrian bridge in a village in rural Bolivia | | |
| 53 | Johri and Sharma, 2012 | T4 | | L1 | Summer research experiences, courses, independent studies, and trips to sites in India. | REU^^[[15]](#footnote-15)^^ | | | Write their thoughts and survey | | | S, T |  | | Improving Immunization and teaching compound interest to semi/illiterate women in rural India | | |
| 54 | Kazmer et al., 2007 | T1 | | L1 | Introduction to engineering |  | | | Survey | | | S, A, R | Students and community partners | | Conventional Structured Bridge Design Project, Technology Exhibition, and project about generation,  consumption, and/or conservation of energy | | |
| 55 | Kilmartin and McCarrick, 2010 | T1 | | L3 | Electrical & Electronic Engineering |  | | | Focus group sessions | | | S, A | Students, faculty and community partners | | To work with clients who have various forms of physical disabilities. | | |
| 56 | Lens and Dewoolkar, 2017 | T2 | | L4 | capstone senior design course |  | | | Written semi-guided reflections on their experience | | | S, A, R | Students | |  | | |
| 57 | Love et al., 2014 | T1 | | L1 | Engineering Design course |  | | | Some questions | | | A, R | Students | | several experiential  service-learning projects | | |
| 58 | Lucena et al., 2010 | Description of “Engineering to Help” (ETH) project and recommendations on where and how to use it in engineering curricula. | | | | | | | | | | | | | | | |
| 59 | Maalouf and Alroomi, 2017 |  | | L1, L4 |  |  | | | Questionnaire^^[[16]](#footnote-16)^^ | | | A, R | Students | | Not specified | | |
| 60 | Mahler et al., 2007 | T4 | | L6 |  | Purdue’s Fall Space Day (PFSD) outreach program | | | Survey | | | S, A, P | Students | | To provide K-12 students with an informal STEM  education experience | | |
| 61 | McCormick et al., 2010 |  | | L4 | Evaluation of sustainability in engineering education | | | | | | | | | | | | |
| 62 | McCormick et al., 2008a | T4 | |  | Summer activity |  | | | Pre-Travel, Post-Travel, Post-Post-travel, and daily surveys | | | S, T, A | Students | | EWB project involving a Green Building Initiative in Ecuador. | | |
| 63 | McCormick et al., 2008b |  | |  | General overview of SL assessment |  | | | Open-ended questions for students and for group assessment, and rubrics | | | S, T, A | Students | |  | | |
| 64 | McLean et al., 2018 | T4 | | L1 | mechanical engineering design course |  | | |  | | |  |  | | Projects in with a local elementary school | | |
| 65 | Meadows and Jarema, 2006 | T1 | | L1 | Introduction to Engineering |  | | | Survey | | | S, T, A | Students | |  | | |
| 66 | Mehta and Enger, 2004 |  | | L1 |  |  | | | The Academic Profile of the Educational Testing Service^^[[17]](#footnote-17)^^ | | | K, S, T, R | Students | |  | | |
| 67 | Mehta et al., 2007 | T1 | | L6 | Engineering Clinics |  | | | Survey | | | K, S, T, R | Students | | Fish Hatchery for a community on the Cheyenne River Reservation | | |
| 68 | Miles et al., 2005 |  | |  | Manufacturing Systems |  | | |  | | | K, T | Students and community partners | | To determine needs and interest for a nonprofit manufacturer | | |
| 69 | Mowry, 2008 | This paper briefly described the characteristics and necessary boundary conditions for the project: a hospital in Tanzania. Specific attention is given to the evaluation process and selection of the alternative-energy based power systems that are needed for sustaining the hospital, the student service-learning work performed in support of this project, the engineering challenges, and the requirements for achieving long-term independent sustainability | | | | | | | | | | | | | To develop and sustain a modern hospital in Tanzania | | |
| 70 | Najmr et al., 2018 | T3 | | L2 to L5 | Chemistry |  | | | Attendance and Participation,  Lab Leadership,  Workbook and Notes, Discussions and Reflections | | | K, S, T, R | Students | | Developing and teaching  chemistry experiments for a secondary school audience | | |
| 71 | Newbolds et al., 2017 | This paper focuses on the use of critical reflection methodologies in an engineering service-learning course. Two different methods of critical analysis: classroom discussions and critical reflection papers. | | | | | | | | | | | | | | | |
| 72 | Oakes and Thompson, 2004 | T1, T3 | | L1 | Engineering Problem  Solving and Computer Tools, English or Communication, and seminars |  | | | | | Survey | S, A, R | Students | | Several projects | | |
| 73 | Oakes and Thompson, 2005 | T1 | | L1 | Engineering Problem Solving and Computer Tools. | EPICS | | | | | Interim reports for students and a survey for community partners |  | Students and community partners | | Several projects | | |
| 74 | Oakes et al., 2001 |  | |  |  | EPICS | | | | | Structure and Phases of EPICS Projects, including the different phases of Finding Project Partners, assembling project teams, etc. | | | | | | |
| 75 | Ocif and Marshall-Goodell, 1996 | T4 | |  |  | Women in Science and Engineering (WISE) program | | | | |  | S, T, R | Students | | Outreach programs with elementary/secondary schoolchildren | | |
| 76 | Osgood et al., 2013 |  | | L1 |  |  | | | | |  | A | Community partners^^[[18]](#footnote-18)^^ | | A need from a remote village in Kenya (designed charcoal presses to convert agricultural waste into charcoal briquettes more efficiently than the current process.) | | |
| 77 | Pagano et al., 2007 | T1, T3 | | L1 | Introduction to Engineering, Service-Learning Engineering Design I and II |  | | | | | Pre- and post-survey | K, T, A | Community partners (high schools students) | | To design a working prototype of a demonstration kit for a local high school science teacher | | |
| 78 | Paterson, 2010 |  | |  |  | Developing Global Scientists and Engineers (DGSE) program | | | | | Mixed methods  assessment plan^^[[19]](#footnote-19)^^ | K, S, T, A | Students | | Research projects that ultimately could create technologies or services designed to improve living conditions in Tanzania | | |
| 79 | Paterson et al., 2009 | A summit on measuring the Impacts of Project-Based Service Learning on Engineering Education | | | | | | | | | | | | | | | |
| 80 | Pierce and Yochum, 2010 | T1 | | L2, L3 | Technology and Society: A Regional Perspective | | |  | | | Final exam, rubric, and survey | K, S, T, R, P | Students | | The design and fabrication of specialized tooling and fixtures to assist developmentally-disabled employees at a local light-assembly plant | | |
| 81 | Pierrakos and Pappas, 2010 | T3 | | L2 |  | | |  | | | Survey, open-ended questions | K, S, T, A | Students | | Designing a device to enable  a professor (and also the client) with cerebral palsy the ability to strengthen his lower and upper body as well as to exercise outdoors | | |
| 82 | Pike, 2009^^[[20]](#footnote-20)^^ |  | | L1 |  | | | Freshman Interest Group (FIG) program | | | Pretest-posttest designs | S, A | Students | |  | | |
| 83 | Pines, 2007 | T4 | | L2 | Engineering by Design | | |  | | | Written report and oral presentation | S, A | Students | | A construction of a water distribution system in some villages in India, including the installation of a solar ground water pump | | |
| 84 | Pizziconi et al., 2010 |  | |  |  | | | Partnership, Pathway, and Pipeline for Engineering Education (P3E2) Project | | | Several instruments^^[[21]](#footnote-21)^^ | S, A | Students and teachers | | A collaboration between the Engineering faculty and a High School and local community members (to engage high school students in the full spectra of STEM-based learning) | | |
| 85 | Portsmore and Swenson, 2012 | T4 | | L5, L6 |  | | | W-STOMP (Women and Student Teacher Outreach Mentorship  Program) | | | Surveys including Draw an Engineer Test (DAET) | A, R | Community partners (high schools students) | | K-12 students engagement in engineering across multiple sources (especially for female engagement) | | |
| 86 | Rahimi, 2008 | Author describes an intensive project-based service learning program, including the community and project Description, partnerships, technical aspects, etc. | | | | | | | | | | | | | Developing a Sustainable Water Distribution and Filtration Project in Rural Honduras | | |
| 87 | Reynaud et al., 2013 | T1 | | L5 | Introduction to Engineering I, Community based Engineering Design Project I and II, etc. | | | SLICE program | | | Surveys^^[[22]](#footnote-22)^^ and reflection exercise | A, P | Students | | Engineering kits for classrooms in Peru, Experimented with homemade solar heaters, etc. | | |
| 88 | Roberts et al., 2007 | It includes a plan to revamp the curriculum in order to better prepare students to take a leading role in improving the infrastructures. The revised curriculum creates a focus on infrastructure topics and the built environment. | | | | | | | | | | | | | Evaluation of infrastructure components in local communities using direct observation, producing a “local” infrastructure report card. | | |
| 89 | Rodriguez et al., 2014 | T1, T2 | | L6 | Several courses (Not specified) | | | Engineering, Education Innovation Center  (EEIC) program | | | Survey, final reports and personal journals | A, S, P | Students | | Projects (in Honduras) varied from water quality/water distribution and alternative energy solutions to agricultural innovations, site assessment and community education. | | |
| 90 | Ropers-Huilman et al., 2005 |  | | L1 | Biological engineering design course | | |  | | | Survey^^[[23]](#footnote-23)^^ | A, R | Students | |  | | |
| 91 | Salam et al., 2019 | T1 | |  | Technopreneurship and Product Development | | |  | | | Online questionnaire and semi-structured interviews^^[[24]](#footnote-24)^^ | K, S, T, A | Students | | Several projects | | |
| 92 | Sanger, 2006 | T1 | | L4 | Project management | | |  | | | A reflection paper and project implementation | K, T, P | Students | | To plan and execute the move of Foothills Industries Inc. (for persons with disabilities to enter the workforce) into their newly constructed facility | | |
| 93 | Schaffer et al., 2007 |  | | L2, L3, L4 | Several courses (not specified) | | |  | | | Survey | K, S, T, A^^[[25]](#footnote-25)^^ | Students | | To design and construct an exhibit for a local science museum that focused on space travel, and to design three different solutions on traffic problems for schools within a local school district. | | |
| 94 | Schaffer et al., 2010 |  | | L5, L6 | Students have completed one  (IPRO)^^[[26]](#footnote-26)^^ course and are enrolled in another to be considered a subject matter  expert (SME) | | | IPRO program | | | Survey^^[[27]](#footnote-27)^^ and reflection | K, S, T, A^25^ | Students | |  | | |
| 95 | Sevier et al., 2012 | T1 | | L1 | Consumer Product Analysis, Manufacturing, and Circuits | | |  | | | Survey^^[[28]](#footnote-28)^^ | K, S, T, A^^[[29]](#footnote-29)^^, P | Students | | A door opener device for a person who has limited use of his hands and arms, and an education assistant for a student at a local high school who has cognitive disabilities and is easily distracted, etc. | | |
| 96 | Stockman et al., 2017 | The study focused on how engineers working on cross-disciplinary design teams use artefacts to communicate and how those artefacts affect design decision making. | | | | | | | | | | | | | | | |
| 97 | Suvannatsiri et al., 2015 | T4 | | L4 | A foundation engineering course | | |  | | | Survey | K, T | community members^^[[30]](#footnote-30)^^ | | To design, construct and test a model of an existing early warning system with simulation of debris flow in a context of a landslide. | | |
| 98 | Swan and McCormick, 2009 | T1 | | L2, L3, L4 | Several courses (not specified) | | |  | | | Survey^^[[31]](#footnote-31)^^ and rubric | S, T, A, P | Students (individual and group assessment)^^[[32]](#footnote-32)^^ | | Efforts with student chapter of EWB, as well as with K-12 outreach efforts through Center for Engineering Education and Outreach (CEEO). | | |
| 99 | Swan and Veti, 2003 | T1 | | L1 | “Waste Not, Want Not”,  an introductory engineering course | | |  | | |  | S, A, P | students | | The evaluation of the reuse options for recovered municipal solid wastes (MSW) for a number of communities. | | |
| 100 | Swan et al., 2007 | T1 | | L6, L5 | Several courses (not specified) | | |  | | | Presentations (oral and written) by team members |  | Students | | Projects in Ecuador, El Salvador, Ghana, and Tibet | | |
| 101 | Swan et al., 2014 | The paper is about the development of community engagement in engineering education and possible research endeavors that can be taken to analyze its potential impact. | | | | | | | | | | | | | | | |
| 102 | Thompson et al., 2005 | T1 | | L1 | Engineering Problem  Solving and Computer Tools | |  | | | | Survey | A, R | | Students | | Several projects | |
| 103 | Thompson and Oakes, 2006 | T1 | | L1 | Engineering Problem Solving and Computer Tools | |  | | | | Surveys and open ended questions | A | | Students and community partners (high schools students) | | Outreach programs for K-12 students | |
| 104 | Tsang et al., 1996 | T1 | | L1 | Introduction to Mechanical Engineering | |  | | | | Survey^^[[33]](#footnote-33)^^ | S, A | | all parties involved in the project | | Projects with middle-school students | |
| 105 | Tsang, 2001 | General paper about Service-Learning in Engineering | | | | | | | | | | | | | | | |
| 106 | Tucker et al., 2013 | Analysis the reasons for faculty interest in learning through service activities | | | | | | | | | | | | | | | |
| 107 | Underwood, 2013 | T1 | | L6 | Engineering Project I to IV | | Integrated Projects Curriculum (IPC), a multi-year plan | | | | Portfolio material and rubrics | K, T | | Students | | Several projects in different locations | |
| 108 | Underwood, 2017 | T1 | | L6 | Engineering Project and Seminar courses | |  | | | |  |  | |  | | Several projects in different locations | |
| 109 | Vernaza et al., 2012 |  | | L6 |  | |  | | | | Survey | A | | Students | | Several projects | |
| 110 | Wang et al., 2012 | T1 | | L1 | Engineering Design and Analysis | |  | | | | Survey based on the ABET criteria and the National Academy of Engineering’s (NAE) ten criteria. | A, S, R, P | | Students | | K-12 service learning through outreach teaching activities | |
| 111 | Whitman and Mason, 2013 | T3 | |  | Service Learning in Engineering | |  | | | | Journal, reflections, presentations, homework | K, S, T, A | | Students | | two types: 1) mentoring or 2) leading the design and build of a LEGO Robotics course. | |
| 112 | Widmann et al., 2011 | T1 | | L6 | Introduction to Mechanical Engineering, Philosophy of Design, Introduction to Design, Intermediate Design, and Senior Design Project | |  | | | |  |  | |  | | Design of adapted devices for persons with disabilities | |
| 113 | Wiggins et al., 2011 |  | | L1, L4, L5 |  | |  | | | | Survey | S, A | | Students | |  | |
| 114 | Williams et al., 2009 |  | | L1 |  | | ROXIE^^[[34]](#footnote-34)^^ | | | | Survey |  | | Students | |  | |
| 115 | Williams, 2002 | About the role of engineers in society and students’  sense of civic responsibility through instruction in communication as part of curricular revision | | | | | | | | | | | | | | | |
| 116 | Won et al., 2017 |  | | L2, L3, L4 | 6-week summer session (between freshmen and sophomore year) | | BOOST program (Bridge Opportunities Offered for the Sophomore Transition) | | | | Survey^^[[35]](#footnote-35)^^ | S, A | | Students | | Projects with two non-profit organizations and a public elementary school | |
| 117 | Wu and Luo, 2018 | This research investigated effective pedagogical approaches and experimented with experiential and project based learning | | | | | | | | | | | | | | | |
| 118 | Zoghi and Pinnell, 2005 | T1 | | L1 | Introduction to Engineering Design (L1, T1), Engineering Design and Appropriate Technology^^[[36]](#footnote-36)^^ | | EPICS and ETHOS programs | | | |  |  | |  | | Wheels for Kids activity, kidslearn project, improvement of quality of life for disabled citizens, etc. | |
| 119 | Zoltowski et al., 2013^^[[37]](#footnote-37)^^ | T2, T3 | | L1 | Service-learning engineering design team courses | |  | | | | Survey^^[[38]](#footnote-38)^^ | A (ethics) | | Students | |  | |
| 120 | Zoltowski et al., 2014 | T4 | | L6 | Summer activity for a group of 13 students from a variety of majors | | EPICS | | | | Survey and final reflection | S, T, A | | Students | | to make a camp more accessible to children with physical disabilities | |

**References**

Canney, N. E., and Bielefeldt, A. R. (2012). “Engineering students’ views of the role of engineering in society,” in *2012 at the ASEE Annual Conference* (San Antonio, TX).

Chan, C. K. Y. (2012). Assessment for community service types of experiential learning in the engineering discipline. *Eur. J. Eng. Educ.* 37, 29–38. doi: 10.1080/03043797.2011.644763

Chrispeels, H.E., Chapman, J. M., Gibson, C.L., and Muday, G.K. (2019). Peer teaching increases knowledge and changes perceptions about genetically modified crops in Non–Science major undergraduates. *CBE Life Sci. Educ.* 18:2, ar14. doi: 10.1187/cbe.18-08-0169

Cowan, D. (2008). “A client-based assessment tool for service learning projects,” in *2008 at the ASEE Annual Conference* (Pittsburgh, PA).

Ding, S. (2008). “Cognitive mapping in Service Learning and civic engagement in a studio course with an ADA accessible project,” in *2008 at the ASEE Annual Conference* (Pittsburgh, PA).

Dixon, G. (2010). “An exploration of promotion and tenure policies related to the scholarship of engagement and outreach at engineering and technology programs within the US,” in *2010 at the ASEE Annual Conference* (Louisville, KY).

Fang, F. C., and Pines, D. (2007). Enhancing transportation engineering education with computer simulation. *Int. J. Eng. Educ.* 23, 808–815. doi: 10.1061/(ASCE)EI.1943-5541.0000038

Gilbert, D.J. (2014). Social work and engineering collaboration: forging innovative global community development education. *J. Soc. Work Educ.* 50:2, 292-304. doi: 10.1080/10437797.2014.885263

Gleixner, S. H., Casey, K., Tuberty, J. T., Latic, S., Backer, P. R., and Allen, E. (2013). “EXCEED: Excellence in your engineering education summer transition program,” in *2013 at the ASEE Annual Conference* (Atlanta, GA).

Goggins, J. (2012). Engineering in communities: learning by doing. Campus-Wide *Inf. Syst.* 29:4, 238-250. doi:10.1108/10650741211253831

Hayden, N. J., Rizzo, D. M., Dewoolkar, M. M., Neumann, M. D., Lathem, S., and Sadek, A. D. E. L. (2011). Incorporating a systems approach into civil and environmental engineering curricula: effect on course redesign, and student and faculty attitudes. *Adv. Eng. Educ.* 2, 1–27. Available online at: https://bit.ly/3wc5UkB.

Jansson, P. M., Heston, W., Molner, R., Murphy, J., and Tomkiewicz, P. (2005). “Undergraduate service learning: campus photovoltaic system siting, design, and permitting,” in *2005 at the ASEE Annual Conference* (Portland, OR).

Jeffers, A. E., Beata, P. A., and Strassmann, B. I. (2014). “A qualitative study to assess the learning outcomes of a civil engineering service learning project in Bolivia,” in *2014 at the ASEE Annual Conference* (Indianapolis, IN). doi: 10.18260/1-2-19986

Johri, A., and Sharma, A. (2012). “Learning from working on others’ problems: case study of an interdisciplinary project-based global service- learning program,” in *2012 at the ASEE Annual Conference* (San Antonio, TX).

Kilmartin, L., and McCarrick, E. (2010). “A case study of enhancing learning outcomes for undergraduate electronic/computer engineering students through a service learning based project module,” in *2010 at the IEEE Transforming Engineering Education: Creating Interdisciplinary Skills for Complex Global Environments* (Dublin), 1–18. doi: 10.1109/TEE.2010.5508846.

Maalouf, S., and Alroomi, A. (2017). “Reversing the hierarchy of causation and effect in civil engineering and construction management courses,” in *2017 ASEE Annual Conference and Exposition* (Columbus, OH). doi: 10.18260/1-2-27816

McCormick, M., Swan, C., and Matson, D. (2008a). “Reading between the lines: Evaluating self assessments of skills acquired during an international service learning project,” in *2008 ASEE Annual Conference and Exposition* (Pittsburgh, PA).

Mahler C., Broughton A., and Caldwell B. (2007). “Fall space day–an educational outreach and professional development program model,” in *2007 ASEE Annual Conference & Exposition* (Honolulu, HI)

Mehta, Y., Jansson, P.M., and Dorland, D. (2007). “Learning through the design of a fish hatchery for a community on the Cheyenne river reservation-an EWB service learning project,” in *2007 ASEE Annual Conference & Exposition* (Honolulu, HI).

Mowry, G. (2008). “A model for developing a sustainable hospital in Tanzania,” in *2008 ASEE Annual Conference & Exposition* (Pittsburgh, PA).

Oakes, W., and Thompson, M. (2005). “Institutionalizing service-learning into a first-year engineering curriculum,” in *2005 ASEE Annual Conference and Exposition* (Fayetteville, AR).

Osgood, L., and Johnston, C. R., and Trivett, A. (2013). “A community partner’s role during a first-year service learning project,” in *2013 ASEE Annual Conference and Exposition* (Atlanta, GA). doi: 10.18260/1-2-19040

Pagano, P., Rossman, A., Vasilnek, K., Aller, B., Kline, A., Tsang, E., et al. (2007). “First-year experience and beyond: Using the engineering design process to support learning and engineering skill development,” in *2007 ASEE Annual Conference and Exposition* (Honolulu, HI).

Pierce, R., and Yochum, H. (2010). “Engaging engineering students in a design-based service learning course emphasizing connections between technology and society,” in *2010 ASEE Annual Conference and Exposition* (Louisville, KY). doi: 10.18260/1-2-16759

Pierrakos, O., and Pappas, E. (2010). “Special session: assessing students’ learning outcomes during a complex and real-world problem-based service learning (PBSL) project in a sophomore engineering design course,” in *2010 ASEE Annual Conference and Exposition* (Louisville, KY). doi: 10.18260/1-2-16209

Pike, G. R. (2009). Assessment measures: assessing program outcomes in the absence of random selection for participation. *Assess. Update* 21, 11–13.

Pines, D. (2007). “Ac 2007-965: gauging student interest in a design for developing communities courses at the University of Hartford,” in *2007 ASEE Annual Conference and Exposition* (Honolulu, HI).

Pizziconi, V., Haag, S., Ganesh, T., Cozort, L., Krause, S., Tasooji, A., et al. (2010). “The P3E2 project: the introduction, implementation and evaluation of engineering design integrated across the middle school curriculum,” in *2010 ASEE Annual Conference & Exposition* (Louisville, KY).

Rahimi, M. (2008). “Ac 2008-785: Key experiences in developing a sustainable water distribution and filtration project in rural Honduras: a new paradigm in “service learning,” in *2008 ASEE Annual Conference and Exposition* (Pittsburgh, PA).

Rodríguez, C. G.M., Gutiérrez Soto,M., Dzwonczyk, R.,Merrill, J. A., Greene, H. L., and Cater, M. (2014). “Application of sustainable solutions in international service-learning engineering projects,” in *2014 ASEE Annual Conference and Exposition* (Indianapolis, IN). doi: 10.18260/1-2-20079

Sanger, P. (2006). “2006-1484: Service learning projects as platforms for an undergraduate project management course,” in *2006 ASEE Annual Conference and Exposition* (Chicago, IL). doi: 10.18260/1-2-865

Suvannatsiri, R., Santichaianant, K., and Murphy, E. (2015). Learning by teaching: Undergraduate engineering students improving a community’s response capability to an early warning system. *Eng. Educ.* 40, 95–113. doi: 10.1080/03043797.2014.928671

Swan, C., Gute, D., Matson, D., and Durant, J. (2007). “International community-based projects and engineering education: the advisor’s viewpoint,” in *2007 ASEE Annual Conference and Exposition* (Honolulu, HI).

Swan, C., Paterson, K., Bielefeldt, A., Johri, A., and Olds, B. (2014). “Community engagement in engineering education as a way to increase inclusiveness,” in *Cambridge Handbook of Engineering Education Research*, ed A. Johri (Cambridge: Cambridge University Press), 357–372. doi: 10.1017/CBO9781139013451.023

Thompson, M., Oakes, W., and Bodner, G. (2005). “A qualitative investigation of a first-year engineering service-learning program,” in *2005 ASEE Annual Conference and Exposition* (Fayetteville, AR).

Underwood, H. R. (2017). “Formalizing experiential learning requirements in an existing interdisciplinary engineering curriculum,” in *2017 ASEE Annual Conference and Exposition* (Columbus, OH).

Widmann, J. M., and Self, B. P., Slivovsky, L. A., and Taylor, J. (2011). “Motivating design and analysis skills acquisition with the infusion of adapted physical activity projects throughout a mechanical engineering curriculum,” in *2011 ASEE Annual Conference and Exposition* (Vancouver, BC). doi: 10.18260/1-2-18858

Williams, C. B., Terpenny, J. P., and Goff, R. M. (2009). “Designing a service-learning design project for a first-year engineering course,” in *ASME 2009 International Design Engineering Technical Conferences and Computers and Information in Engineering Conference* (San Diego, CA), 631–639. doi: 10.1115/DETC2009-87091

Won, D., Menezes, G. B., Sharif, A. A., Ragusa, G., and Pacheco-Vega, A. (2017). “Boosting engineering identity of rising sophomore engineering majors through service learning based bridge program,” in *2017 ASEE Annual Conference and Exposition* (Columbus, OH).

Wu, W., and Luo, Y. (2018). “Technological and social dimensions of engaging lower division undergraduate construction management and engineering students with experiential and project-based learning,” in *Construction Research Congress 2018* (New Orleans, LA), 97–107.

1. “Community Service Attitudes Scale” (CSAS): an instrument for measuring college students’ attitudes about community service. The CSAS items were based on Schwartz’s (Schwartz & Howard, 1982; Shiarella, 2000) model of altruistic helping behavior. [↑](#footnote-ref-1)
2. Research Experience for Undergraduates (REU), Summer Multicultural Access to Research and Training (SMART), Discovery Learning (DL) Apprenticeship and Multicultural Engineering Program (MEP) programs. [↑](#footnote-ref-2)
3. Survey based on: (1) ABET (a)-(k) criteria, and (2) the paper by Seymour et al. (2004). [↑](#footnote-ref-3)
4. Based on the validated Engineering Professional Responsibility Assessment (EPRA). They also calculate the total volunteer frequency score (VFS). [↑](#footnote-ref-4)
5. Written and oral presentation (16%), an initial written workplan (4%), written alternatives assessment (draft; 25%), and final alternatives assessment and preliminary design (written and oral presentation; 45%), a reflection on their SL experience (10%). [↑](#footnote-ref-5)
6. Based on the validated Engineering Professional Responsibility Assessment (EPRA). [↑](#footnote-ref-6)
7. An introductory nonmajors biology course for Non–Science Major Undergraduates [↑](#footnote-ref-7)
8. A client-based survey of service learning that is based on End-of-Program Survey from Shinnamon et al. (1999); and Health Professions Schools in Service to the Nation (HPSISN). They also include SL program assessment for directors/faculty survey. [↑](#footnote-ref-8)
9. Civic Mindedness Survey and Civic Minded Graduate Reflection Prompt, using the methodology ‘response shift bias’ (asks students to take a survey, then immediately afterwards take it again). [↑](#footnote-ref-9)
10. The SL project is mandatory and contributes 20% of the final course grade. The course has other activities different from SL. [↑](#footnote-ref-10)
11. Habitat is a self-help housing organization devoted to build “simple, decent, and affordable” housing for low income families. ADA: Americans with Disabilities Act. [↑](#footnote-ref-11)
12. Multivariate analysis of variance (MANOVA) with a Tukey HSD (Honestly Significant Difference) post hoc analysis was used to determine if any significant differences were present between student groups for both surveys. [↑](#footnote-ref-12)
13. Excellence in Your Engineering Education Summer Transition Program. [↑](#footnote-ref-13)
14. Self-report instruments go beyond simply asking students if they learned a topic or achieved an outcome and ask them to reflect on their knowledge and behaviors [↑](#footnote-ref-14)
15. REU (Research Experiences for Undergraduates) program. [↑](#footnote-ref-15)
16. Questionnaire with mathematical notations (∞: Agree or High, μ: Neutral or Average and ∅: Disagree or Low) were used in lieu of typical scales that are usually found in most questionnaires (such as extremely agree to extremely disagree). [↑](#footnote-ref-16)
17. The Academic Profile is a well established, well-researched instrument for measuring several aspects of general education, including college-level reading, critical thinking, writing, and mathematics in the context of material from the humanities, social sciences, and natural sciences. [↑](#footnote-ref-17)
18. The following criteria were identified as critical in determining whether a community organization representative (COR) developed a positive or negative view of the SL experience: • whether objectives were clearly aligned and defined; • level of communication and preparation between the COR and faculty member; • amount of student motivation and conduct; and • whether the COR’s problem was resolved.

    This paper will examine the advantages as well as the inconveniences experienced by a COR while participating in a SL project. [↑](#footnote-ref-18)
19. The mixed methods assessment plan consists of surveys, reflection statements, journaling, a wellness indicator, the Intercultural Development Inventory© (IDI©), and project reports. The IDI© assesses intercultural competence. It is a 50-item questionnaire available online that can be completed in 15–20 minutes. [↑](#footnote-ref-19)
20. Pike includes a discussion about assessment of SL programs, when considering non-random assignment of students to programs. [↑](#footnote-ref-20)
21. The following instruments have been used: 1) a STEM content and attitude survey for students, 2) the “Draw an Engineer” assessment instrument for students and teachers, 3) a combined survey on Design, Engineering, Technology (DET) and Tinkering self-efficacy for teachers, 4) a mid-year assessment by teachers to determine if the project was meeting its goals, 5) an assessment of students and teachers based on an activity designed from materials included in the magazine "Engineering, Go for It", and 6) a reflection about the project by students and their parents. [↑](#footnote-ref-21)
22. The survey is included. [↑](#footnote-ref-22)
23. Students participated in a survey and in focus groups to explore how well the service-learning project helped them to meet EC 2000 a-k objectives. [↑](#footnote-ref-23)
24. Cross-Disciplinary Functioning Survey (CDFS). [↑](#footnote-ref-24)
25. Assessment of cross-disciplinary team learning (CDTL). [↑](#footnote-ref-25)
26. The Inter-professional Projects (IPRO) course offers students experience in tackling a semester-long open-ended challenge as part of an interdisciplinary team (<https://ipro.iit.edu/>). [↑](#footnote-ref-26)
27. Includes a statistical analysis using ANOVA. [↑](#footnote-ref-27)
28. Specifically designed to measure ABET criteria [↑](#footnote-ref-28)
29. Attention, Relevance, Confidence, and Satisfaction (ARCS model) are assessed. [↑](#footnote-ref-29)
30. They are assessed about landslides, the early warning system for landslides (EWSLS) and math skills [↑](#footnote-ref-30)
31. Explain in (McCormick et al., 2008b) [↑](#footnote-ref-31)
32. They use the professional skills instrument (Group Assessment Form) allowed group members to evaluate themselves and each other. [↑](#footnote-ref-32)
33. Including some questions from classroom climate inventory, the Michigan organizational assessment questionnaire and the job satisfaction questionnaire. [↑](#footnote-ref-33)
34. ROXIE includes numerous projects for the community, directed at finding solutions to problems around their operation or economic viability that involve working with limited resources, conserving the earth’s resources, and minimizing environmental impact. [↑](#footnote-ref-34)
35. Authors use an engineering creativity and innovation index: Engineering Creativity and Propensity for Innovation Index (ECPII) (Ragusa, 2011). [↑](#footnote-ref-35)
36. Engineering Design and Appropriate Technology includes a six to sixteen week summer service-learning internship, the capstone design course (L4) where design projects are “real-life” applications with team of students responsible for different aspects of design, and a series of seminars for sophomores through seniors each semester, etc. [↑](#footnote-ref-36)
37. This paper addresses the ways in which instructors can utilize an engineering ethical reasoning instrument in engineering class or lab sessions. [↑](#footnote-ref-37)
38. Authors define the Engineering Ethical Reasoning Instrument (EERI) for assessment of individual students’ ethical reasoning. [↑](#footnote-ref-38)
